# Cytogenetic analysis of two locariid species (Teleostei, Siluriformes) from Iguatemi River (Parana River drainage) in Brazil

**DOI:** 10.3897/CompCytogen.v9i1.8804

**Published:** 2015-03-10

**Authors:** Carlos Alexandre Fernandes, Diandra Soares Alves, Zaira da Rosa Guterres, Isabel Cristina Martins-Santos

**Affiliations:** 1 State University of Mato Grosso do Sul, BR 163-Km 20.2-CEP: 79980-000, Mundo Novo, MS, Brazil; 2 Department of Cell Biology and Genetics, State University of Maringá, Avenida Colombo 5790, 87020-900, Maringá, PR, Brazil; 3 Grupo de Estudo em Ciências Ambientais e Educação (GEAMBE)

**Keywords:** Pterygoplichthini, Chromomicin A_3_, DAPI, Chromosomal evolution, Fishes

## Abstract

Fishes of the Loricariidae family, known as “cascudos”, constitute an endemic group in Neotropical freshwaters. In this study, were cytogenetically examined two species of Loricariidae (*Pterygoplichthys anisitsi* Eigenmann & Kennedy, 1903 and *Farlowella amazonum* (Günther, 1864) belonging to Hypostominae and Loricariinae subfamilies respectively) from Iguatemi River. Our study provide the first description regarding C-band and fluorochromic analysis in *Farlowella amazonum*. In *Farlowella amazonum*, diploid number was 58 chromosomes, with single Ag-NOR and heterochromatic blocks in centromeric regions of some chromosomes and large subtelomeric blocks were evidenced on the long arm of the pair 27, being this region CMA_3_^+^/DAPI^-^. The *Pterygoplichthys anisitsi* showed diploid number equal 52 chromosomes, with single Ag-NOR and heterochromatic blocks in centromeric and telomeric regions of some chromosomes and conspicuous large telomeric blocks on the long arm of the pair 10, being this region CMA_3_^+^/DAPI^-^. The results show that karyotype formula is nonconservative in *Pterygoplichthys anisitsi* and *Farlowella amazonum*.

## Introduction

Fishes of the Loricariidae family, known as “cascudos”, constitute an endemic group in Neotropical freshwaters and are morphologically characterised by the body covered by several rows of plates and a ventral mouth with lips forming a sucker (Graça and Pavanelli 2007). Currently, this family includes 887 valid species in seven subfamilies: Hypoptopomatinae, Loricariinae, Hypostominae, Neoplecostominae, Lithogeninae, Delturinae, and Ancistrinae (Eschmeyer and Fong 2014). In earlier phylogenetic studies, Ancistrinae (as a tribe Ancistrini) was considered as a tribe in the family Hypostominae along with Hypostomini, Rhinelepini, Pterygoplichthini, and Corymbophanini (Armbruster 2004). According to the latter author, the Pterygoplichthini is composed by genera and species groups: *Pterygoplichthys* Gill, 1858, *Hemiancistrus annectens* group, being that *Liposarcus* Günther, 1864, and *Glyptoperichthys* Weber, 1991 are recognized as synonyms of *Pterygoplichthys*.

Available cytogenetic data for Hypostominae subfamily show that the diploid number ranges from 2n = 34 in *Ancistrus cuiabae* Knaack, 1999 (Mariotto et al. 2009) and Anscistrus sp. purus INPA-25625 (Oliveira et al. 2009) to 2n = 84 in *Hypostomus* sp. 2 (Cereali et al. 2008). The tribe Hypostomini is one of the most studied from the cytogenetic point of view, with wide variation in chromosome number ranging from 2n = 54 in *Hypostomus plecostomus* (Linnaeus, 1758) (Muramoto et al. 1968, cited by Artoni and Bertollo 2001) to 2n = 80 in *Hypostomus* sp. E (Artoni and Bertollo 1996). On the other hand, for tribe Pterygoplichthini cytogenetic studies are scarce, which all species presenting a diploid number of 52 chromosomes, as observed in *Pterygoplichthys joselimaianus* (Weber, 1991) (Oliveira et al. 2006), *Pterygoplichthys anisitsi* Eigenmann & Kennedy, 1903 (cited as *Liposarcus anisitsi* – Alves et al. 2006), *Pterygoplichthys multiradiatus* (Hancock, 1828) (cited as *Liposarcus multiradiatus* – Alves et al. 2006) and *Pterygoplichthys gibbiceps* (Kner, 1854) (cited as *Glyptoperichthys gibbiceps* – Alves et al. 2006).

According to Alves et al. (2003), in Loricariinae subfamily, only some genera as: *Harttia* Steindachner, 1877, *Loricaria* Linnaeus, 1758, *Loricariichthys* Bleeker, 1862, *Rineloricaria* Bleeker, 1862, and *Sturisoma* Swainson, 1838 were analyzed cytogenetically, presenting diploid number ranging of 2n = 36 in *Rineloricaria latirostris* (Boulenger, 1900) (Giuliano-Caetano 1998) to 2n = 74 in Sturisoma cf. nigrirostrum Fowler, 1940 (Artoni and Bertollo 2001). Specifically, in the genus *Farlowella* Eigenmann & Eigenmann, 1889 (Loricariinae) cytogenetic studies are rare, and restricted to *Farlowella amazonum* (Günther, 1864), which has showed a diploid number of 58 chromosomes (Fernandes et al. 2012).

In the present study, we carried out cytogenetic analyses in *Pterygoplichthys anisitsi* Eigenmann & Kennedy, 1903 and *Farlowella amazonum* (Günther, 1864). Besides Giemsa, we used C-band, Ag-NOR, CMA_3_ and DAPI techniques to evaluate cytogenetically the species. Our results provide the first description of C-band and analysis with fluorochromes in *Farlowella amazonum* and these results were used to discuss some aspects of the chromosome evolution in the Hypostominae and Loricariinae subfamilies.

## Materials and methods

**Figure 1.**
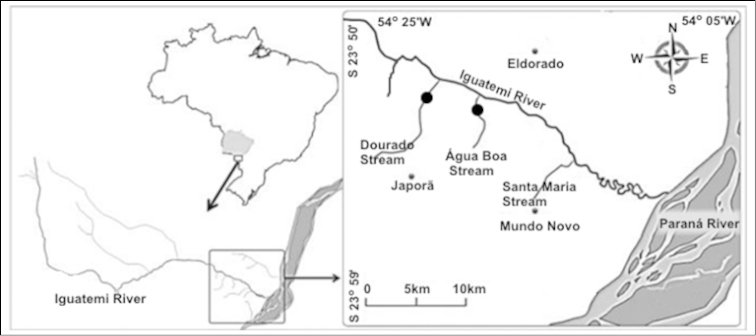
Localization of the Água Boa and Dourado streams from Iguatemi River Basin where specimens were captured. Dark circles indicate the sampled points.

Four (2 males and 2 females) specimens of *Farlowella amazonum*, from Dourado stream and four (2 males and 2 females) specimens of *Pterygoplichthys anisitsi*, from Água Boa stream were analyzed. Dourado (23°51'04,9"S and 54°25'13,9"W) and Água Boa (23°50'16,65"S and 54°20'55,54"W) streams are tributaries of right bank of the Iguatemi River, Mato Grosso do Sul State, Brazil (Fig. 1).

The fishes were identified and deposited in the State University of Mato Grosso do Sul, Mundo Novo. The experiments followed ethical conducts, and before evisceration process, the fishes were anesthetized by an overdose of clove oil (Griffiths 2000). Metaphasic chromosomes were obtained from anterior kidney cells using the air-drying technique (Bertollo et al. 1978). Analysis of the C-positive heterochromatin (C-bands) followed the basic procedure of Sumner (1972), with some minor adaptations. The NORs were detected by means of silver nitrate staining (Ag-NORs), according to Howell and Black (1980). Regions rich in GC and AT were detected by fluorochromes chromomycin A_3_ (CMA_3_) and DAPI (4’6-diamidino-2-phenylindole) respectively, according to procedure proposed by Schmid (1980).

About 30 metaphases were analyzed for each specimen and those with better chromosome morphology were used in the karyotype analysis. The chromosomes were classified as metacentric (m), submetacentric (sm), subtelocentric (st) and acrocentric (a) according to their arm ratio (Levan et al. 1964). For the determination of the fundamental number (FN), or number of chromosome arms, the m, sm and st chromosomes were considered as bearing two arms and the acrocentric chromosomes only one arm.

## Results

**Figure 2.**
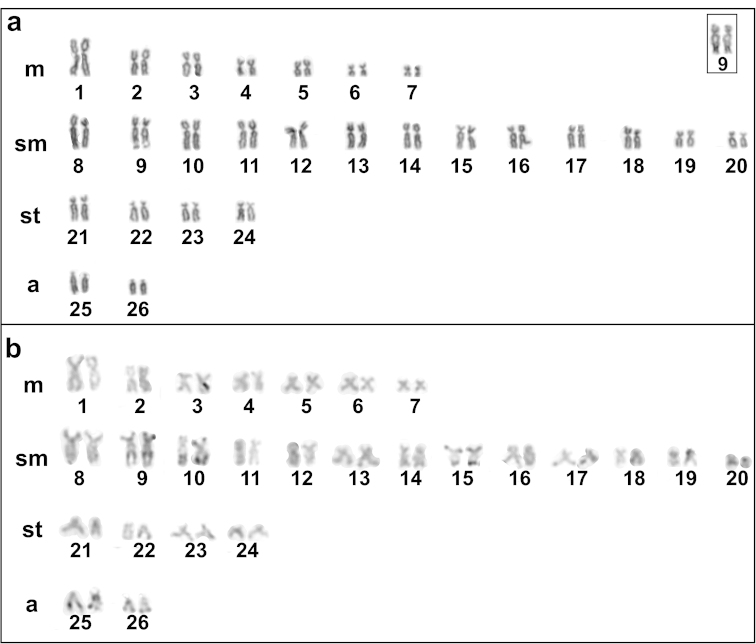
Karyotypes stained with Giemsa (**a**) and C-banding (**b**) of *Pterygoplichthys anisitsi* from Água Boa stream. Box: pair 9, bearing the NOR.

**Figure 3.**
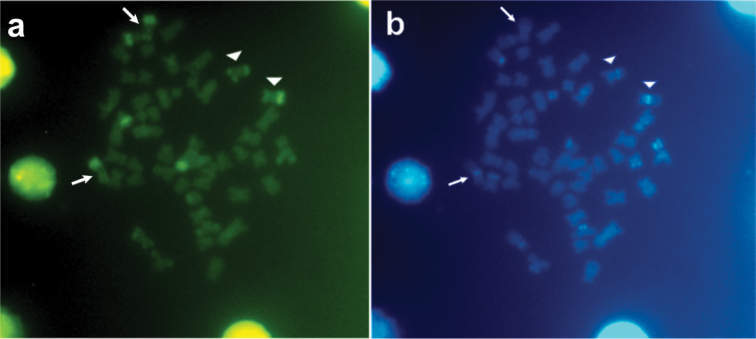
Metaphases of *Pterygoplichthys anisitsi* stained with (**a**) Chromomycin A_3_ and (**b**) DAPI. Arrows indicate pair 10 and arrows head indicate pair 9 (NOR-bearing).

*Pterygoplichthys anisitsi* presented a modal diploid number of 52 chromosomes in males and females, distributed in 14m+26sm+8st+4a, with a FN of 100 in both sexes (Fig. 2a). The Ag-NORs were located in a subtelomeric position on the long arm of the acrocentric pair 9, coinciding with a secondary constriction (Fig. 2a). Heterochromatic blocks evident at telomeric regions were observed in the pairs 17, 25 and 26. Also, evident bitelomeric markings were present in the pairs 10 and 15, and conspicuous large telomeric blocks were present on the long arm of the pair 10 and interstitial blocks were evidenced on the long arm pair 9, adjacent to the Ag-NOR region (Fig. 2b). CMA_3_ staining produced fluorescent signals in the telomeric regions of some chromosomes, fluorescent signals bitelomeric in the pairs 9, 10 being conspicuous the fluorescent signals on the long arm of the pair 10 (Fig. 3a). DAPI staining proved adjacent markings to CMA3^+^ and fluorescent signals in the telomeric regions of some chromosomes, indicating that those regions are rich in AT (Fig. 3b). In addition, DAPI staining revealed pale regions corresponding to telomeric regions on the long arm of the pair 10, coinciding with CMA_3_^+^ region, confirming that those regions are poor in AT.

**Figure 4.**
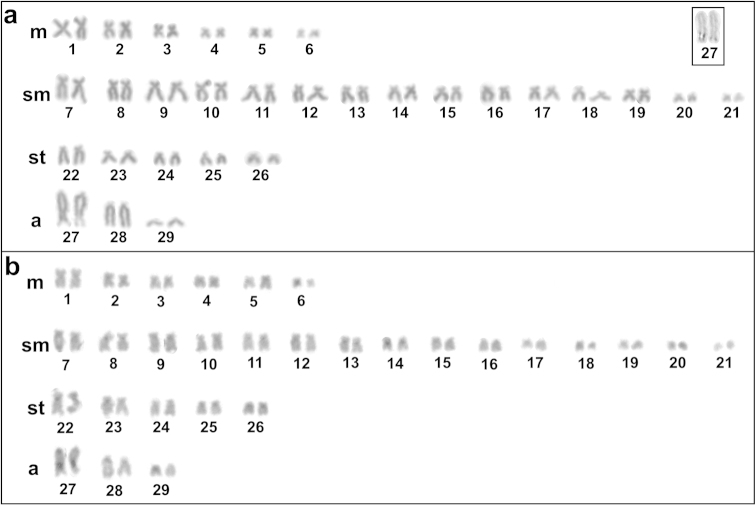
Karyotypes stained with Giemsa (**a**) and C-banding (**b**) of *Farlowella amazonum* from Dourado stream. Box: pair 27, bearing the NOR.

*Farlowella amazonum* presented a modal diploid number of 58 chromosomes in males and females, distributed in 12m+30sm+10st+6a, with a FN of 110 in both sexes (Fig. 4a). The Ag-NORs were located in a telomeric position on the long arm of the pair 27, coinciding with a secondary constriction and with size heteromorphism (Fig. 4a). Heterochromatic blocks at centromeric regions were observed in the pairs 8, 11, 13, 14, 22, 23 and 27 and large subtelomeric blocks were evidenced on the long arm of the pair 27, adjacent to the NOR region (Fig. 4b). CMA_3_ staining produced fluorescent signals on the long arm of the pair 27, corresponding to Ag-NOR region with size heteromorphism. This staining also evidenced fluorescent signals on the end portion of the long arm of the pair 28, indicating that these regions are rich in GC (Fig. 4a). DAPI staining revealed only pale regions corresponding to the CMA_3_ marked regions, confirming that those regions are poor in AT (Fig. 4b).

## Discussion

Diploid chromosome numbers ranged from 2n = 34 to 2n = 84 in Hypostominae (Cereali et al. 2008; Mariotto et al. 2009). However, when different tribes of Hypostominae are separately analyzed, it is possible to notice that this variation is not widespread among them. If Pterygoplichthini is concerned, the diploid number of 52 chromosomes found in *Pterygoplichthys anisitsi* is also observed in *Pterygoplichthys joselimaianus* from Lago Quatro Bocas (Oliveira et al. 2006), *Pterygoplichthys anisitsi* from Miranda and Tietê Rivers (Alves et al. 2006), *Pterygoplichthys multiradiatus* from Orinoco River (Alves et al. 2006), and *Pterygoplichthys gibbiceps* from Orinoco River (Alves et al. 2006). In spite of this trend towards conservatism in relationship to diploid number, differences in karyotype constitution in Pterygoplichthini, especially regarding the number of acrocentric chromosomes are observed. *Pterygoplichthys anisitsi* presented four acrocentric chromosomes, as well as, populations of *Pterygoplichthys anisitsi* from Tietê River (Alves et al. 2006) and Preto River (Artoni et al. 1999), while *Pterygoplichthys anisitsi* from Miranda River presented sixteen acrocentric chromosomes (Alves et al. 2006). On the other hand, *Pterygoplichthys joselimaianus* (Oliveira et al. 2006), *Pterygoplichthys multiradiatus* (Alves et al. 2006) and *Pterygoplichthys gibbiceps* (Alves et al. 2006) do not present acrocentric chromosomes. Thus, all these populations of *Pterygoplichthys* studied differ in their karyotypic formulae, with intra- and interspecific variations, suggesting the occurrence of chromosome rearrangements, such as pericentric inversions, that can alter the morphology of the chromosomes without changing the diploid number.

The intraspecific variation in *Pterygoplichthys anisitsi* may be explained by the distribution of species already analyzed. Thus, *Pterygoplichthys anisitsi* populations of the Iguatemi (present study), Tietê and Preto Rivers (Alves et al. 2006) that belong to the same watershed (Upper Paraná River Basin) showed no differences in the number of acrocentric chromosomes, except the *Pterygoplichthys anisitsi* population of the Miranda River (Alves et al. 2006) that belongs to another basin (Paraguai River basin) showed a number of acrocentric chromosomes different. Therefore, the geographical isolation of *Pterygoplichthys anisitsi* populations (Paraná River Basin and the Paraguai River Basin) may have facilitated the establishment of karyotypic variation. The *Pterygoplichthys multiradiatus* and *Pterygoplichthys gibbiceps* populations (Alves et al. 2006) are also geographically isolated (Orinoco River basin) of the *Pterygoplichthys anisitsi* populations. Thus, the lack of gene flow between them could favor the establishment of distinctive changes in each sample, putatively resulting in a speciation process.

With respect to nucleolar organizer regions, the present study detected two active NORs in *Pterygoplichthys anisitsi*. Others species previously analyzed of tribe Pterygoplichthini as *Pterygoplichthys joselimaianus* (Oliveira et al. 2006), *Pterygoplichthys anisitsi* (Artoni et al. 1999, Alves et al. 2006), *Pterygoplichthys multiradiatus* (Alves et al. 2006; Alves et al. 2012) and *Pterygoplichthys gibbiceps* (Alves et al. 2006) also present this same pattern, with subterminal markings, but with differences in location (long or short arm) and type of NOR-bearing chromosome. According to Oliveira and Gosztonyi (2000) the condition of single Ag-NORs in subterminal location is the possible basal condition for the Siluriformes. Thus, in Pterygoplichthini all species analyzed presented single Ag-NORs in subterminal location, suggesting the maintenance of this basal condition. The presence of single Ag-NORs also is described in Ancistrini (Alves et al. 2006; Mariotto et al. 2011; Cardoso et al. 2013), which is coincident with Ag-NORs results for Pterygoplichthini. Furthermore, diploid number of 52 chromosomes is predominant in Ancistrini (Kavalco et al. 2005, Alves et al. 2006). This statement reinforces the sister-group relationship between Pterygoplichthini and Ancistrini hypothesized by Armbruster (2004).

In *Pterygoplichthys anisitsi*, the present study revealed that Ag-NOR is compositionally GC-rich and heterochromatin blocks adjacent NOR region are compositionally AT-rich (Fig. 6a). In *Hypostomus* sp. B heterochromatin blocks adjacent NOR regions also are compositionally AT-rich (Artoni and Bertollo 1999). In addition, heterochromatin blocks visualized by C-banding correspond to majority of the chromosomes marked with CMA_3_, suggesting that these constitutive heterochromatins are compositionally GC-rich. This can be clearly observed in the pair 10, which was C-band^+^, CMA3^+^ and DAPI^-^. The DAPI standing produced fluorescent signals adjacent to CMA3^+^ markings, and also in the telomeric regions of some chromosomes, probably in those with heterochromatic blocks CMA3^-^, showing that these regions are compositionally AT-rich. In Pterygoplichthini, there are few studies focused on constitutive heterochromatin, which are restricted to *Pterygoplichthys anisitsi* from Preto River (Artoni et al. 1999), confirming the pattern of compositionally GC-rich regions. The constitutive heterochromatin GC-rich are commonly found in Loricariidae (Artoni et al. 1999; Artoni and Bertollo 1999; Kavalco et al. 2004; Rubert et al. 2008). However, constitutive heterochromatin AT-rich as described for *Pterygoplichthys anisitsi* is rare event among fishes, being reported mainly among some Hypostominae species (Artoni and Bertollo 1999).

**Figure 5.**
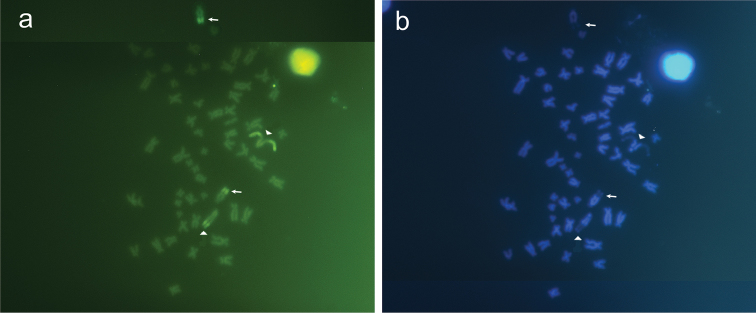
Metaphases of *Farlowella amazonum* stained with (**a**) Chromomycin A_3_ and (**b**) DAPI. Arrows indicate pair 28 and arrows head indicate pair 27 (NOR-bearing).

**Figure 6.**
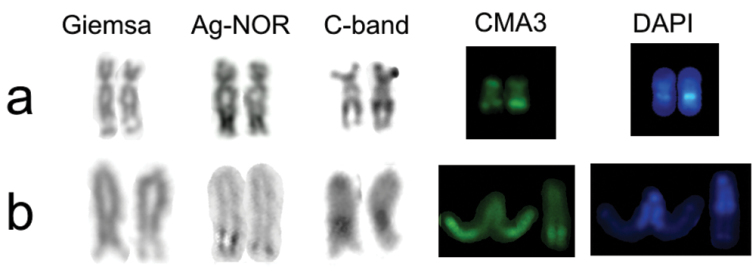
NOR-bearing chromosomes of *Pterygoplichthys anisitsi* (upper row – **a**) and *Farlowella amazonum* (lower row – **b**).

With 239 species, Loricariinae is second largest subfamily of Loricariidae in species number (Eschmeyer and Fong 2014). Despite this high species number, cytogenetic descriptions are restricted to the karyotypes of few genera. *Farlowella amazonum* analyzed in this study had a diploid number of 58 chromosomes, which is coincident with previously described diploid number of a population from Água Boa stream (Fernandes et al. 2012), but with minor karyotype differences, mainly regarding to the number of metacentric and submetacentric chromosomes. The population analyzed here presented 12 metacentric and 30 submetacentric chromosomes, while the population previously described by Fernandes et al. (2012) presented six metacentric and 38 submetacentric chromosomes. Although the two streams are tributaries of the Iguatemi River, which could facilitate gene flow between the two populations of *Farlowella amazonum* and conservation of micro- and macrostructure karyotypic, it is possible that this is not happening, since the differences in numbers metacentric and submetacentric chromosomes between the two populations is significant. Therefore, probably the isolation two populations *Farlowella amazonum* may have facilitated the establishment of karyotypic changes. In this species, the occurrence of chromosome rearrangements, such as pericentric inversions, that can alter the morphology of the chromosomes without changing the diploid number probably occurred in the karyotypic evolution of the group. This inference emphasizes the importance of develop more studies focused on cytogenetic of genus *Farlowella* in order to clear such question.

In *Farlowella amazonum*, were detected single Ag-NORs in a telomeric position on the long arm of the pair 27, corresponding to the same location described in *Farlowella amazonum* from Água Boa stream (Fernandes et al. 2012). Others species previously analyzed of Loricariinae as *Harttia kronei* Miranda Ribeiro, 1908, *Rineloricaria kronei* (Miranda Ribeiro, 1911), *Rineloricaria cadeae* (Hensel, 1868), *Rineloricaria* n. sp., *Harttia loricariformis* Steindachner, 1877 (Alves et al. 2003) and *Harttia punctata* Rapp Py-Daniel & Oliveira, 2001 (Blanco et al. 2014) also presented two NOR-bearing chromosomes, but located on the short arm, except the *Harttia loricariformis* and *Harttia punctata* that presented NOR interstitial on the long arm.

The CMA_3_/DAPI results for *Farlowella amazonum*, which is the first description of literature, showed that Ag-NOR is compositionally GC-rich (Fig. 6b). In *Rineloricaria cadeae*, *Rineloricaria strigilata* (Hensel, 1868) and *Rineloricaria pentamaculata* Langeani & de Araújo, 1994 the Ag-NOR also showed compositionally GC-rich (Maia et al. 2010). On the other hand, *Harttia loricariformis* that presented C-bands conspicuous in the NOR-bearing did not present positive CMA_3_ or DAPI staining heterochromatin (Kavalco et al. 2004). NOR-size heteromorphism among homologues detected in *Farlowella amazonum* by Ag-NOR and confirmed by CMA_3_/DAPI staining, is a common event in fish. According to Phillips et al. (1989), CMA_3_ analysis coupled to silver nitrate has been useful in detecting polymorphism of these sites. The NOR-size heteromorphism may be explained by transposition events or unequal crossing-over in this region.

Regarding to C-band pattern, also inedited for *Farlowella amazonum*, the results revealed weak centromeric markings in some chromosomes and large subtelomeric blocks on the long arm of the pair 27, adjacent to the NOR region. These large heterochromatic blocks showed correlation with CMA_3_ markings, suggesting that these constitutive heterochromatins are compositionally GC-rich (Fig. 6b).

The interstitial position heterochromatic blocks adjacent to the NOR region in *Farlowella amazonum* and *Pterygoplichthys anisitsi* may indicate that heterochromatin dispersive processes, as proposed by (Schweizer and Loidl 1987), are common to the subfamilies Hypostominae and Loricariinae, that revealed to be independent of the heterochromatin compositional, AT-rich in *Pterygoplichthys anisitsi* and GC-rich in *Farlowella amazonum*.

According to Kavalco et al. (2005) in Loricariidae, the diploid number of 54 chromosomes seems to be a plesiomorphic condition. The cytogenetic data obtained in present study for *Pterygoplichthys anisitsi*, as well as, those described in the literature for species of Pterygoplichthini (Artoni et al. 1999, Alves et al. 2006, Oliveira et al. 2006), show that all have 2n = 52 chromosomes. The presence of 2n = 52 chromosomes in Pterygoplichthini is probably an apomorphic characteristic, suggesting the reduction in the diploid number in the ancestor of this tribe. The diploid number of 58 chromosomes in *Farlowella amazonum* also is probably an apomorphic characteristic, suggesting the increase in the diploid number in the ancestor in the *Farlowella*. According to Kavalco et al. (2005) in the subfamily Loricariinae, both centric fusion, centric fissions and pericentric inversions arise as common karyotypic rearrangements.
